# HEPeak: an HMM-based exome peak-finding package for RNA epigenome sequencing data

**DOI:** 10.1186/1471-2164-16-S4-S2

**Published:** 2015-04-21

**Authors:** Xiaodong Cui, Jia Meng, Manjeet K Rao, Yidong Chen, Yufei Huang

**Affiliations:** 1 Department of ECE, University of Texas at San Antonio, TX 78249, USA; 2 Department of Biological Science, Xi'an Jiaotong-liverpool University, Suzhou, 215123, China; 3 Greehey Children's Cancer Research Institute, University of Texas Health Science Center at San Antonio, TX 78229, USA

## Abstract

**Background:**

Methylated RNA Immunoprecipatation combined with RNA sequencing (MeRIP-seq) is revolutionizing the de novo study of RNA epigenomics at a higher resolution. However, this new technology poses unique bioinformatics problems that call for novel and sophisticated statistical computational solutions, aiming at identifying and characterizing transcriptome-wide methyltranscriptome.

**Results:**

We developed HEP, a Hidden Markov Model (HMM)-based Exome Peak-finding algorithm for predicting transcriptome methylation sites using MeRIP-seq data. In contrast to exomePeak, our previously developed MeRIP-seq peak calling algorithm, HEPeak models the correlation between continuous bins in an m^6^A peak region and it is a model-based approach, which admits rigorous statistical inference. HEPeak was evaluated on a simulated MeRIP-seq dataset and achieved higher sensitivity and specificity than exomePeak. HEPeak was also applied to real MeRIP-seq datasets from human HEK293T cell line and mouse midbrain cells and was shown to be able to recapitulate known m^6^A distribution in transcripts and identify novel m^6^A sites in long non-coding RNAs.

**Conclusions:**

In this paper, a novel HMM-based peak calling algorithm, HEPeak, was developed for peak calling for MeRIP-seq data. HEPeak is written in R and is publicly available.

## Background

RNA methylation is an emerging area that studies chemical modifications in the nucleotides of RNAs [1-4]. Such modification in especially coding mRNAs or transcripts has been shown [5,6] or speculated to play a critical role in regulating cellular functions [7-9]. However, the overall mechanism by which mRNA is methylated and the related functions in different contexts including various diseases are still elusive. Deciphering their functions and regulations under various contexts represents a grand challenge facing the biology community.

**Figure 1:**
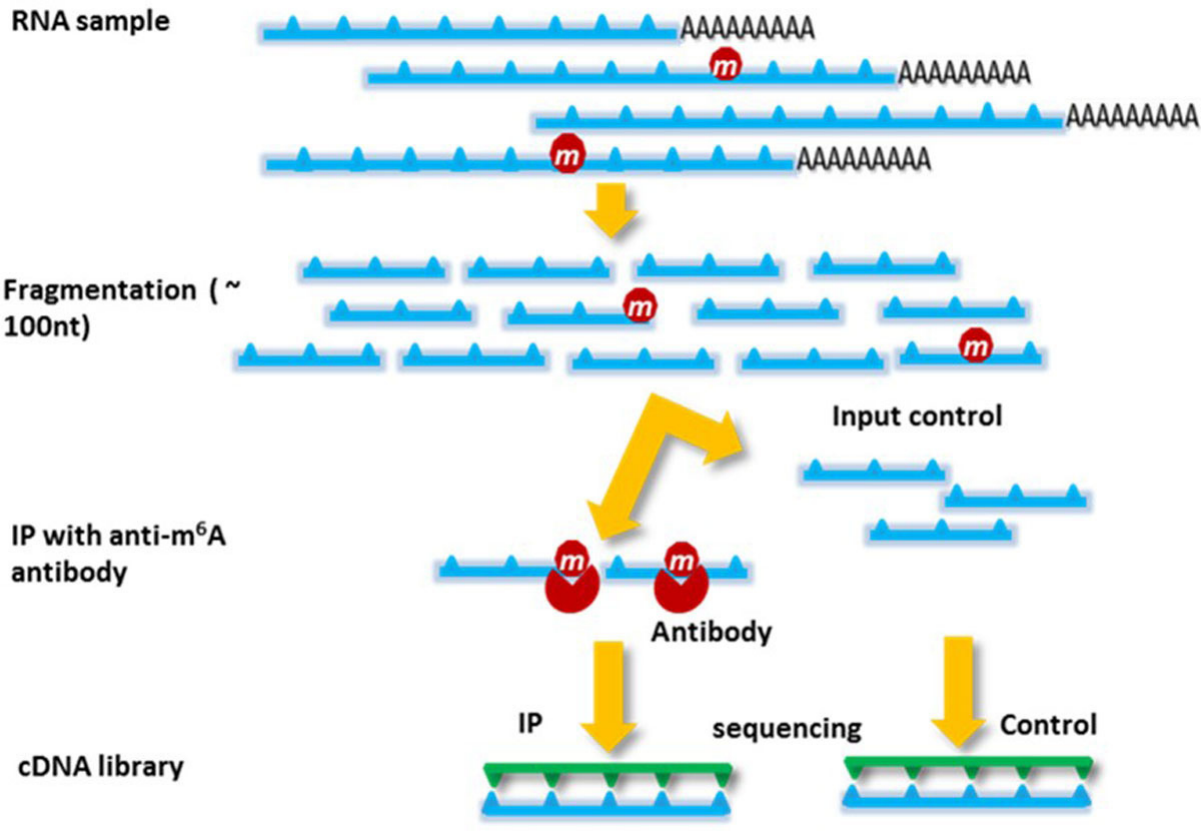
**Illustration of MeRIP-seq**. Total RNAs are collected from cells and fragmented into ~100 nt long. A part of fragmented RNAs is taken as the control sample, which is submitted for sequencing. The remaining part is subject to immunoprecipitation by antibody to isolate the methylated sequences, which are then submitted for sequencing.

The state-of-the-art high throughput technology that enables the detection of RNA methylation in transcriptome is an affinity-based shotgun sequencing approach known as Methylated RNA immunoprecipitation (IP) sequencing (MeRIP-Seq) [2]. MeRIP-Seq was first introduced in recent studies [1,2,10,11] on transcriptome-wide mRNA m^6^A methylation and is a high throughput sequencing assay that is designed for transcriptome-wide survey of RNA epigenetics [6]. As shown in Figure 1, in MeRIP-seq, mRNA is first fragmented before immunoprecipitation with anti-m6A antibody, and then the immunoprecipitated and control mRNA fragments are subject to sequencing. The output includes an IP and a control sample, which measure the immunoprecipitated m^6^A-methylated mRNA reads and the mRNA expression (or RNA-seq measurement), respectively. These paired samples are used to reconstruct the transcriptome-wide m^6^A methylome. While MeRIP-seq has demonstrated high accuracy in identifying the cell-specific transcriptome methylation patterns, as a nascent assay, MeRIP-Seq poses unique bioinformatics challenges that call for novel and sophisticated statistical computational algorithms.

From a biological perspective, MeRIP-Seq can be thought as a combination of two well-studied methods: ChIP-Seq [12-14] and RNA-Seq [15,16]. Like ChIP-seq, reads accumulate around the methylation sites to form *peaks*. Unlike ChIP-seq based measurements for DNA methylation, MeRIP-seq measures mRNA methylation and hence produces read peaks around the methylation sites that span two or more exons. In addition, the control sample of MeRIP-seq measures mRNA expression, which, compared to those in ChIP-Seq, can vary much more drastically in different cells or tissues. Due to these unique features, ExomePeak [17] was developed specifically for peak calling, or methylation site prediction, in MeRIP-seq. Although ExomePeak can perform fairly robust exome-based peak calling, it ignored the dependency of reads, and therefore could either miss true peaks with low intensity or erroneously predict narrow, noisy outliers as true peaks. In this paper, we introduce HEPeak, a novel Hidden Markov model (HMM) for exome-based peak calling algorithm. The test results showed that HEPeak improved both prediction sensitivity and specificity over ExomePeak.

## Methods

### HEPeak pipeline

To address the aforementioned MeRIP-seq issues, HEPeak includes several high-throughput sequencing tools in its pipeline. First, HEPeak utilizes TopHat [18] to align fragmented mRNA reads to the reference transcriptome, allowing short reads to span exon-exon junctions. Next, SAM-tools [19] is applied to exclude the multi-mapping reads and index alignment results. After these pre-processing steps, HEPeak performs HMM-based peak calling on the exons of each gene, where the introns are excluded, to identify the genomic locus of methylation sites. The output result of HEPeak is in BED format, which can be visualized together with input alignments in IGV2.1 [20].

### Exome-based peak calling

The goal of peak calling in MeRIP-seq is to detect regions in transcripts where the read counts in the IP sample is more "enriched" than those in the control sample. Just as with ExomePeak, our previously developed peak calling algorithm for MeRIP-seq, HEPeak performs the peak calling on connected exons of a specific gene, a clear contrast to genome-based ChIP-seq peak calling methods, such as MACS [21]. This projection of genome onto transcriptome effectively circumvents the difficulty due to the ambiguity of isoforms' assignment but it still preserves the convenience of gene-based annotation, making biological interpretation of the prediction straightforward.

### The definition of HMM for MeRIP-seq data

Given a particular mRNA (RefSeq gene), its concatenated exons are first divided into *N *mutually connected bins, whose size is selected as the read length *L*. With respect to the *n_th _*bin, the unknown hidden methylation status is denoted as *z_n _*∈ {1, 2} where 1 represents unmethylation and 2 otherwise. Since a peak likely spans multiple bins, we assume that the methylation status *z_n _*follows a first order Markov chain, whose transition matrix *A *contains entries defined as

(1) $$A_{jk}=P\left(z_{n}=k|z_{n-1}=j\right),\;\;\;\;j,k\in \left\{1,2\right\}$$

where *A_jk _*denotes the probability for the latent variable switching from the status *j *at the (*n *- 1)*_th _*bin to the status *k *at the *n_th _*bin. Here *j*, *k *is the indicator of the hidden state. Additionally, we assume that the initial probability *P*(*z*_l _= 1) = *π *and *P*(*z*_l _= 2) = 1 - *π*.

Next, let *x_n _*denote the read counts in the IP sample and *y_n _*the counts in the control sample, both for bin *n*. We assume that, given the methylation status *z_n_*, these read counts follow the Poisson distribution defined as

(2) $$P\left(x_{n}|t_{n}\right)=Pois\left(M_{IP}\lambda_{IP,z_{n}}\right)$$

(3) $$P\left(y_{n}|t_{n}\right)=Pois\left(M_{ctrl}\lambda_{ctrl}\right)$$

where *M_IP _*and *M_ctrl _*are the total reads (sequencing depth) in the IP and the control samples, respectively and $\lambda_{IP,z_{n}}$ for *z_n _*= 1, or 2 and *λ_ctrl _*are the normalized Poisson rates, respectively. It is worthwhile pointing out that $\lambda_{IP,z_{n}}$ switches according to the status of *z_n_*; on the contrary, *λ_ctrl _*stays the same.

It would be intuitive next to define the relationship between the Poisson rates for the methylated and unmethylated in the IP and the control sample, respectively. However, unlike in ChIP-seq, where this relationship is mostly defined only for the IP sample, defining the relationship for both the IP and the control is non-trivial and model complexity also needs to be assessed to avoid potential difficulties in subsequent inference. To this end, we transform the formulation by observing that, given (2) and (3), the conditional probability of observing *x_n _*in the IP given the total reads in the control as *t_n _*= *x_n _*+ *y_n _*follows the binomial distribution

(4) $$P\left(x_{n}|z_{n},t_{n}\right)=Bino\left(t_{n},p_{z_{n}}\right)$$

where

(5) $$p_{z_{n}}=\frac{M_{IP}\lambda_{IP,z_{n}}}{M_{ctrl}\lambda_{ctrl}+M_{IP}\lambda_{IP,z_{n}}}.$$

Note that $p_{z_{n}}$ for *z_n _*= 1 (or 2) can be considered as the percentage of the mean IP read counts in the combined read counts of the IP and control samples for a bin, when it is unmethylated (or methylated). The distribution (4) effectively combines the reads in the IP and control samples under one model. As such, instead of using (2) and (3), we define (4) as the emission probability of the proposed HMM and work with $p_{z_{n}}$ directly. Doing so avoids modelling and inferring the potentially complex relationships between the rates. Given **X **= {*x*_1_, *x*_2_, *x*_3_,..., *x_N_*}, a set of reads for *N *bins and **Z **= {*z*_1_, *z*_2_, *z*_3_,..., *z_N_*}, the sequence of methylation, we use *γ*(*z*_*n*,*k*_) to denote the marginal posterior distribution of a latent variable *z*_*n *_at state *k*, and *ε*(*z*_*n*-1_, *z*_*n*_) to denote the joint posterior distribution of two successive latent variables, so that

(6) $$\gamma \left(z_{n,k}\right)=p\left(z_{n}=k|X,\theta \right)$$

(7) $$\varepsilon \left({z_{n-1}}_{,j},z_{n,k}\right)=p\left(z_{n-1}=j,z_{n}=k|X,\theta \right).$$

Here, the parameter is defined as $\theta =\left\{A_{k,j}\forall k\forall j;\pi ;p_{k}\forall k\right\}$. Then, the log likelihood for the proposed HMM chain can be expressed as

(8) $$\begin{array}{c} Q=E_{z}\left[\text{ln}P\left(X,Z|\theta \right)\right]=\overset{2}{\underset{k=1}{\sum }}\gamma \left(z_{1,k}\right)\text{ln}\pi_{k}\\ \;+\overset{N}{\underset{n=1}{\sum }}\overset{2}{\underset{j=1}{\sum }}\gamma \left(z_{n,k}\right)\text{ln}P\left(x_{n}|z_{n,k}\right)+\overset{N}{\underset{n=2}{\sum }}\overset{2}{\underset{j=1}{\sum }}\overset{2}{\underset{k=1}{\sum }}\varepsilon \left(z_{n-1,j}z_{n,k}\right)\text{ln}A_{jk} \end{array}$$

**Figure 2:**
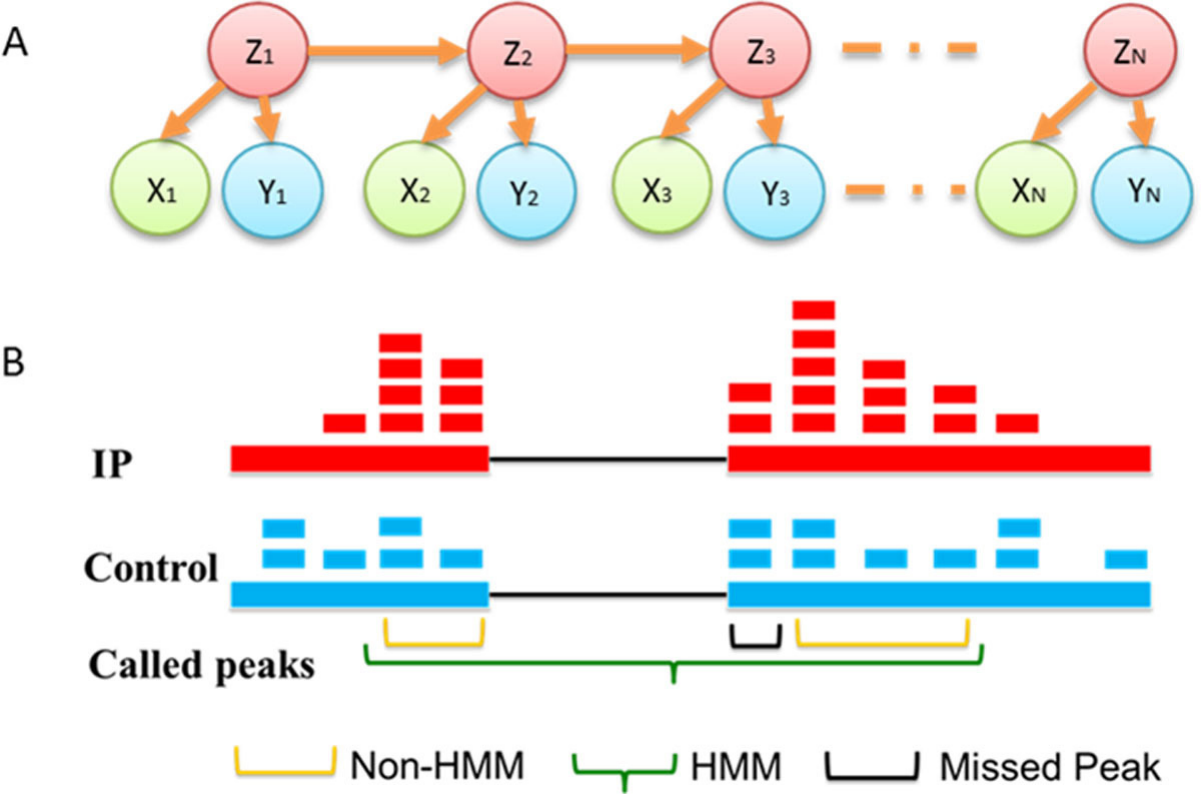
**Illustration of the proposed Hidden Markov model**. **A**. The graphical model of the proposed hidden Markov model. **B**. An illustration of the advantage of the proposed HMM. The region marked by a black bracket would be missed by a non-HMM based algorithm such as exomePeak because the reads do not show enrichment in IP. However, this region is likely part of the peak because it is located in the middle of consecutively enriched regions.

We call this new formulation HEPeak or Hidden Markov Model (HMM)-based Exome Peak finding. The graphical model of HEPeak formulation is shown in Figure 2A. Compared with ExomePeak, HEPeak considers the correlation of the reads between adjacent bins and more accurately models the behaviour of methylated reads in MeRIP-Seq (Figure 2B).

### The EM solution

Given HEPeak, the goal is to call peaks, i.e., predict *z_n_*∀*n*, and at the same time estimate the model parameters: *θ*. To this end, we developed an Expected-Maximization (EM) solution, which performs peak calling and parameter estimation in an iterative fashion. We provide the steps of the EM algorithm in the following. The detailed derivation is included in appendix.

At the *m_th _*iteration, proceed as follows.

**E step**: Given parameter *θ*^(*m*-1)^, estimated at the *m*-1 step, calculate the posterior distribution of the latent variable *P*(**Z**|**X**, *θ*^(*m*-1)^).

(9) $$\gamma \left(z_{n,k}\right)=p\left(z_{n}=k|X,\theta^{\left(m-1\right)}\right)$$

**M step**: Compute and update *π*^(*m*)^, *A_jk_*^(*m*) ^and *p_k_*^(*m*) ^for all *j*, *k *as

(10) $$\pi =\frac{\gamma \left(z_{11}\right)}{\overset{1}{\underset{j=0}{\sum }}\gamma \left(z_{1j}\right)}$$

(11) $$A_{jk}=\frac{\overset{N}{\underset{n=2}{\sum }}\varepsilon \left(z_{n-1,j},z_{n,k}\right)}{\overset{1}{\underset{l=0}{\sum }}\overset{N}{\underset{n=2}{\sum }}\varepsilon \left(z_{n-1,j},z_{n,l}\right)}$$

(12) $$p_{k}=\frac{\overset{N}{\underset{n=1}{\sum }}\gamma \left(z_{n,k}\right)X_{n}}{\overset{N}{\underset{n=1}{\sum }}\gamma \left(z_{nk}\right)\left(X_{n}+Y_{n}\right)}$$

After the EM iteration converges, the model parameter *θ *can be obtained. Given the estimated *θ*, the Viterbi algorithm is applied to maximize the joint likelihood in (8) to obtain the maximum *a posteriori *(MAP) estimate of the methylation status *z_n_*.

### Peak region detection

In order to evaluate the statistical significance of the putative peak regions predicted by the Viterbi algorithm, the log odds ratio of the posterior for the peak state (*z_n _*= 2) over the posterior for the background state (*z_n _*= 1) can be computed as follows

(13) $$\text{PeakScore}\left(z_{n}\right)=\text{log}\frac{p\left(z_{n}=2|X\right)}{p\left(z_{n}=1|X\right)}$$

Briefly, this log-transformed scoring method [22-24] tries to utilize the posterior probability of each bin to assess the confidence of the potential peak region. The potential peak region is defined as consecutive bins predicted by the Viterbi and its PeakScore is calculated as the averaged PeakScores for all the combined bins. Next, PeakScore is assumed to follow a Gaussian distribution with mean (*mean*(PeakScore)) and standard deviation(*std*(PeakScore)) [24], estimated from all the bins. Then, after performing the z transform of PeakScores, a one-sided test for significance of the potential peak region can be conducted and p-value can calculated. Then, the Benjamini-Hochberg method [25] is utilized to correct the multiple testing and compute the False Discovery Rate (FDR).

## Results

### Simulation test

Because we do not have the ground truth for the methylation status in real data, the performance of HEPeak was first validated using a simulated data, where read counts for the IP and the control samples were simulated according to the proposed HEPeak model.

Specifically, a total of 5000 genes, whose lengths were randomly selected from 500 nt to 3k nt, were generated. Reads of each gene in both IP and the control samples were allowed to vary according to the Poisson distribution, where we chose *λ *∈ (5 ~ 20) and assumed it constant for both methylated and unmethylated bins. Additionally, we set *λ_IP _*∈ (*λ_ctrl_*, 100) when methylated and *λ_IP _*= (0, *λ_ctrl_*), when unmethylated, resulting in 14200 peaks generated. The transition matrix *A *was defined as

**Figure 3:**
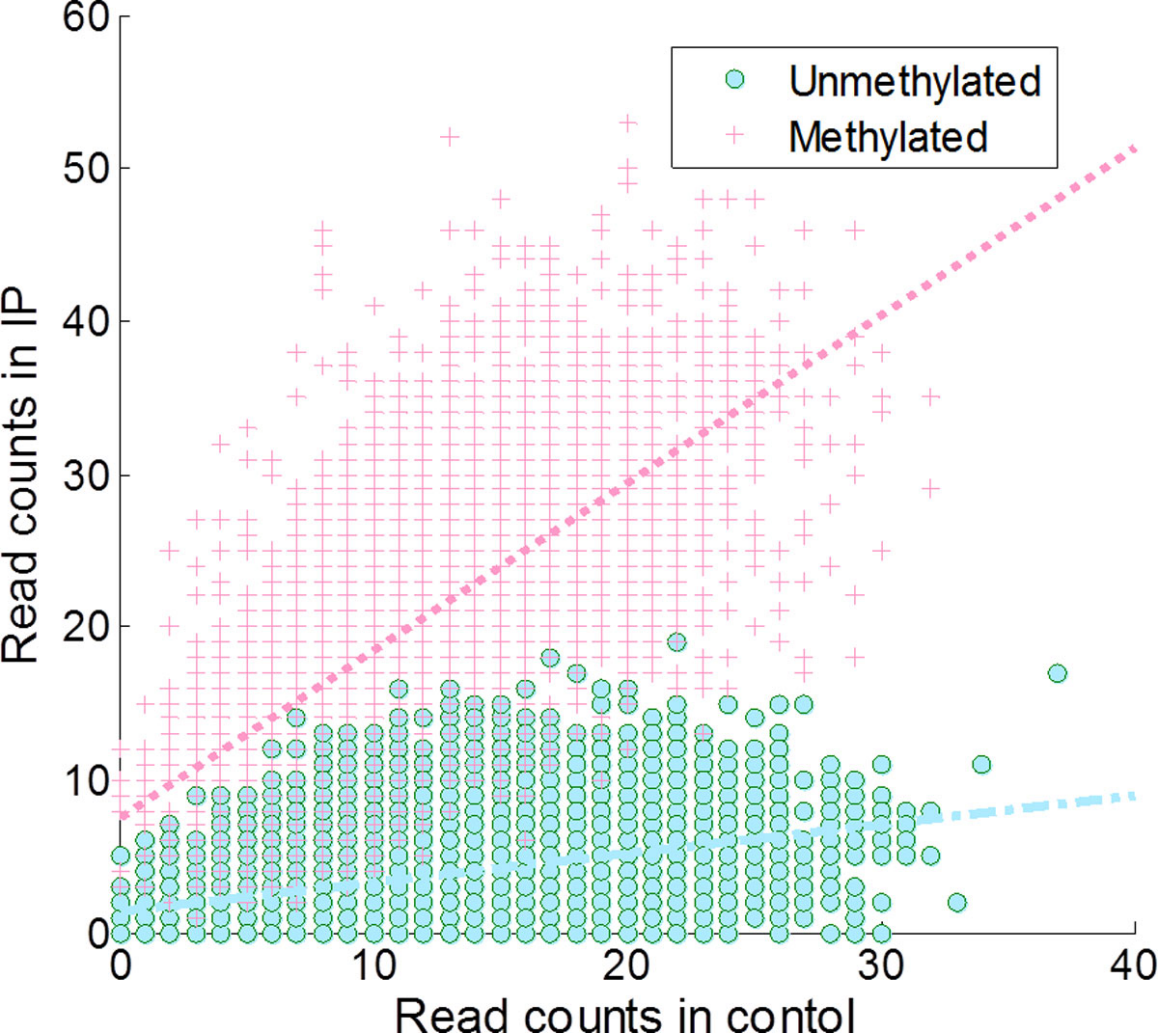
**Scatterplot of simulated MeRIP-seq reads in IP and control samples**. In unmethylated regions, reads were more enriched in the control, while in methylated regions, they were made more enriched in the IP.

$A=\left[\begin{array}{cc} 0.7 & 0.3\\ 0.1 & 0.9 \end{array}\right]$

and the initial probability *π *= 0.2 Note that *A *and *π *were based on the estimates obtained by HEPeak when applied to the real m^6^A data discussed in the next section. Figure 3 showed an illustration of the simulated data. In general, when a bin is methylated, there were more reads in IP than in control; otherwise, there were more reads in control.

**Figure 4:**
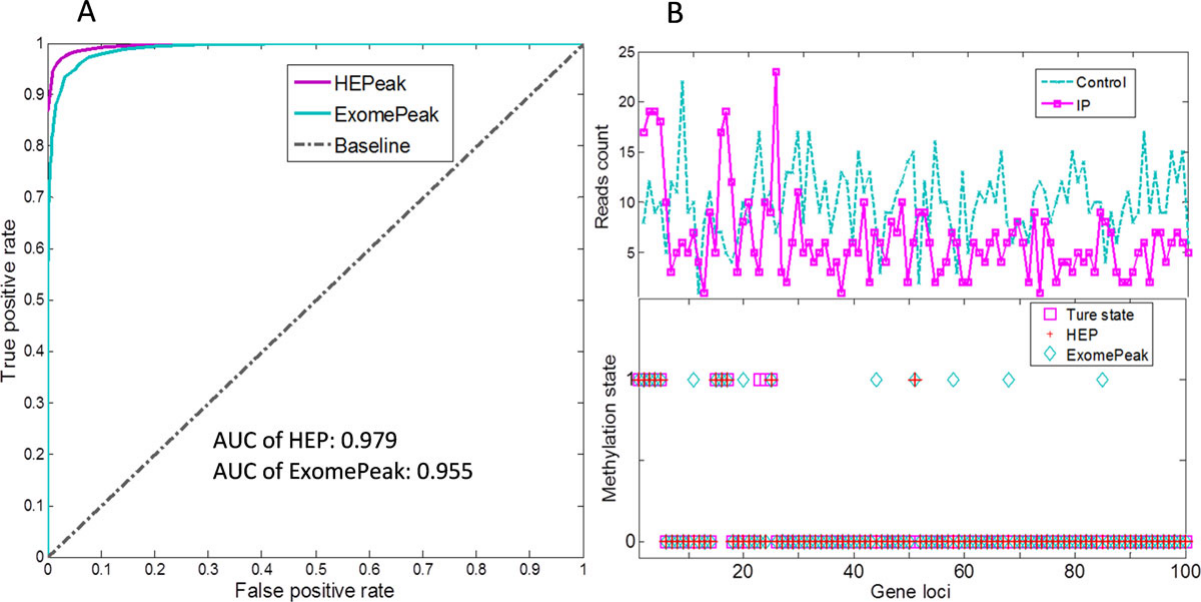
**Simulation results illustrate HEPeak performs better than ExomePeak**. **A**. The ROC curve of HEPeak and exomePeak. **B**. An example of a simulated gene loci, where there are 10 positive and 90 negative peaks. The top panel depicts the simulated read counts and the bottom panel shows the predicted results of HEPeak and exomePeak. exomePeak detects 8 of 10 true positives, with false positive rate 7.78%; while HEPEAK achieved the same sensitivity but made much fewer false positives at about 1.11%.

The receiver operating characteristics (ROC) curve of the peak calling results is shown in Figure 4A and we can see that the ROC curve of HEPeak wraps around that of ExomePeak, which indicates that HEPeak achieves a higher detection sensitivity and specificity. The area under the curve (AUC) for HEPeak is 0.979, which is larger than that of ExomePeak (0.955). As shown in Figure 4B, the read distributions of a simulated gene with 10 bins marked as methylated peaks and 90 bins as unmethylated, the corresponding detection results show that HEPeak can correctly detect 8 out of 10 true peaks, with 1 false positive, while ExomePeak results in 7 false positives to get the same sensitivity.

### Evaluation of HEPeak on real m^6^A MeRIP-seq data

To further validate the accuracy of HEPeak, we applied HEPeak to two m^6^A MeRIP-seq datasets including one from human HEK293T cell line [1] and the other from the mouse midbrain cells [8]. The raw fastq datasets were obtained from Gene Expression Omnibus (GEO accession: GSE29714 and GSE47217). The datasets were preprocessed according to the HEPeak pipeline, where the raw data was first aligned to the reference hg19 and mm10 assembly by TopHat, and then peak calling was performed to predict the transcriptome-wide m^6^A methylation for each dataset. As a comparison, ExomePeak was also applied to these datasets.

**Figure 5:**
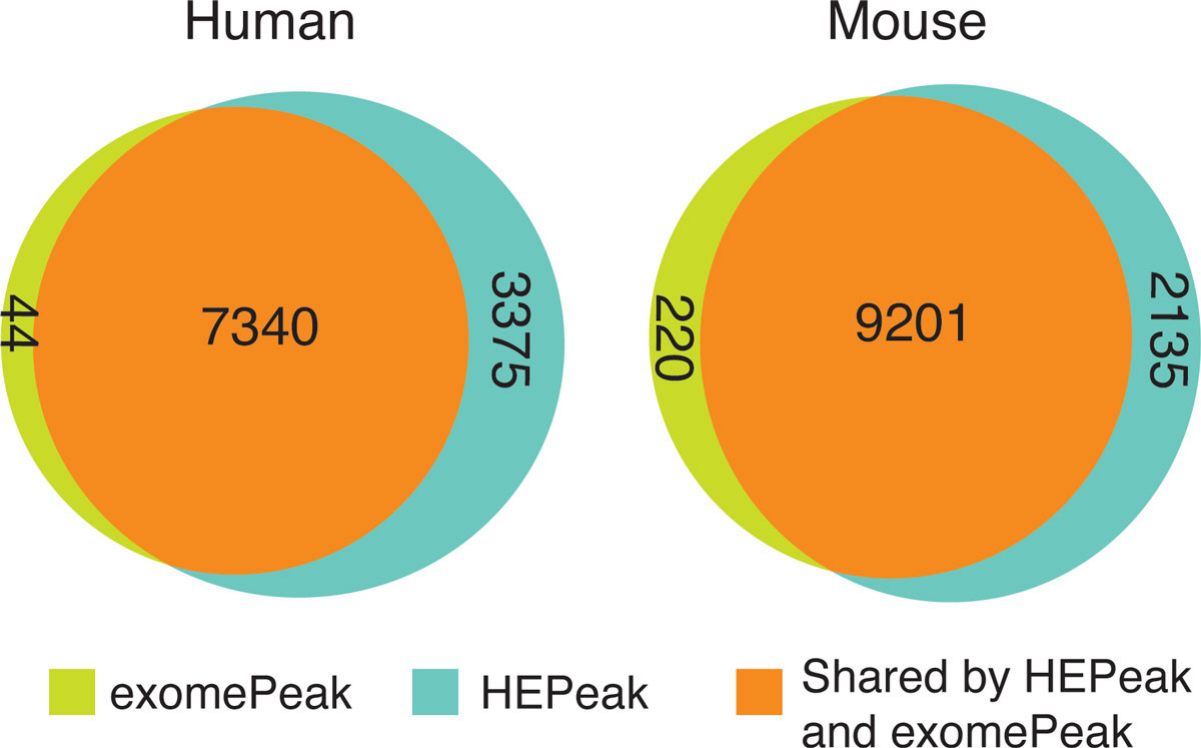
**Methylated genes found in mouse midbrain cell by HEPeak and ExomePeak**. In human cell, exomePeak uniquely found 44 genes being methylated, while HEPeak detected 3375. In mouse, exomePeak found 220 genes being methylated, and HEP reported 2135.

**Figure 6:**
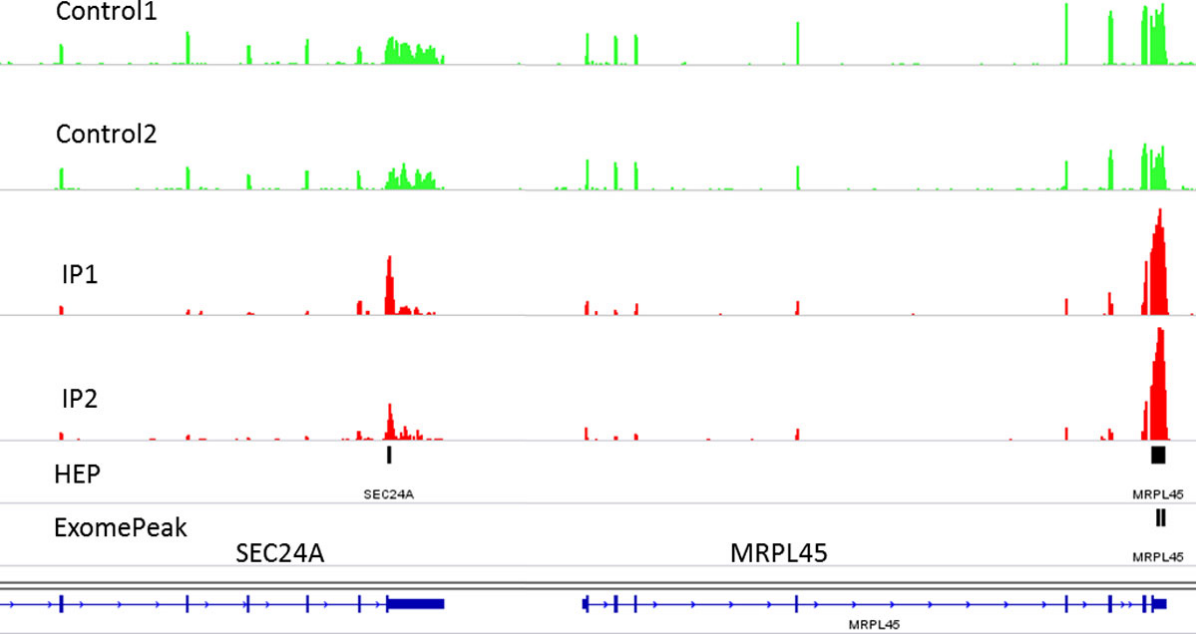
**An example of peak calling results on gene SEC24A and MRPL45**. The top four tracks depict the reads density in control and IP samples respectively. The bottom two tracks show the predicted peaks of HEPeak and exomePeak, where the predicted peak regions are marked by black bars.

A large number of genes were predicted to have m^6^A methylation sites in both human and mouse datasets. For HEK293T dataset, HEPeak identified 24281 peaks on 10715 genes at a FDR < 0.025, whereas ExomePeak (at the default setting) reported 15164 peaks on 7344 genes. Out of all the genes, 7340 genes were predicted to be methylated by both HEPeak and ExomePeak, whereas 3375 genes were predicted only by HEPeak, as opposed to 44 genes uniquely reported by ExomePeak (Figure 5A). For mouse midbrain cells, HEPeak discovered 25138 peaks on 11336 genes (FDR < 0.025); in contrast, ExomePeak detected 19324 peaks on 9421 genes. Among them, 9201 genes were shared by the two algorithms, while HEPeak identified 1915 more genes than ExomePeak (Figure 5B). The above results demonstrate that more potential methylated genes ignored by ExomePeak, can be discovered by HEPeak, which makes use of dependency of consecutive bins and greatly boosts the detection sensitivity. The advantage of HEPeak becomes even clearer if we carefully examined the results in IGV for the two datasets (Figure 6A and Figure 6B). Take HEK293T dataset for example. For gene SEC24A, visual inspection should confirm methylation where read counts in the IP sample show slight enrichment to that in control sample. HEPeak demonstrate a higher sensitivity by utilizing the whole consecutive bins to determine the peak region where reads are greatly enriched compared to other region. For gene MRPL45, both methods found m^6^A methylation sites. However, due to HMM, HEPeak correctly merged the two peaks into one peak.

### HEPeak recapitulates previous reported m^6^A patterns

**Figure 7:**
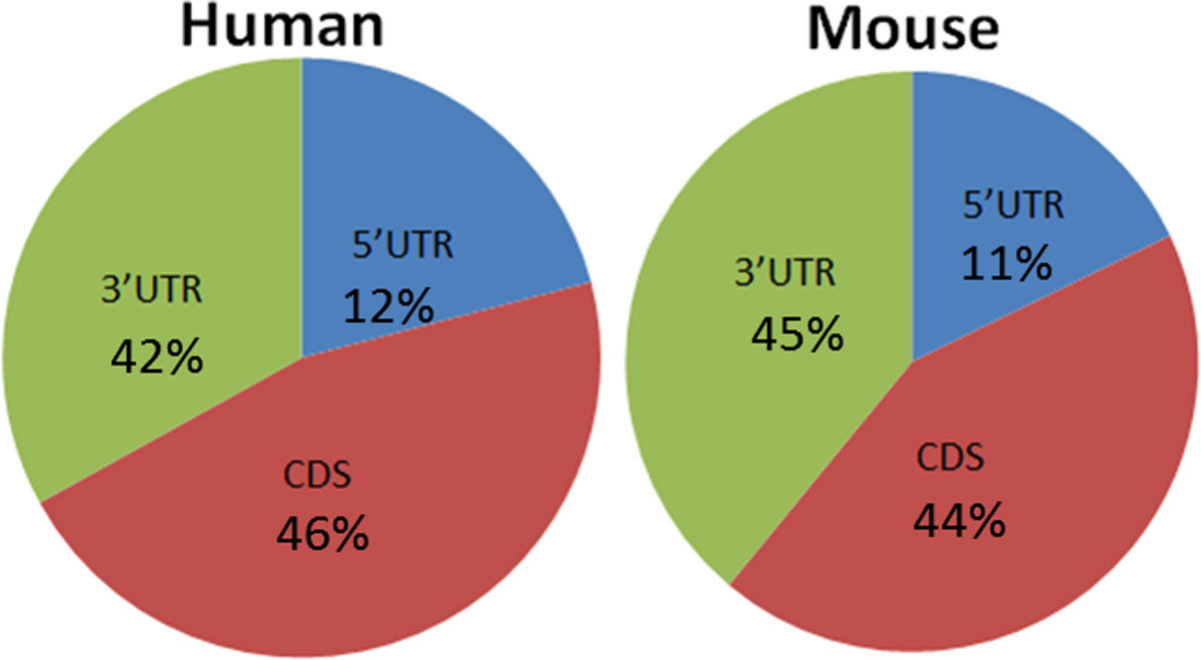
**Proportions of m^6^A occurrence in mRNAs**.

**Figure 8:**
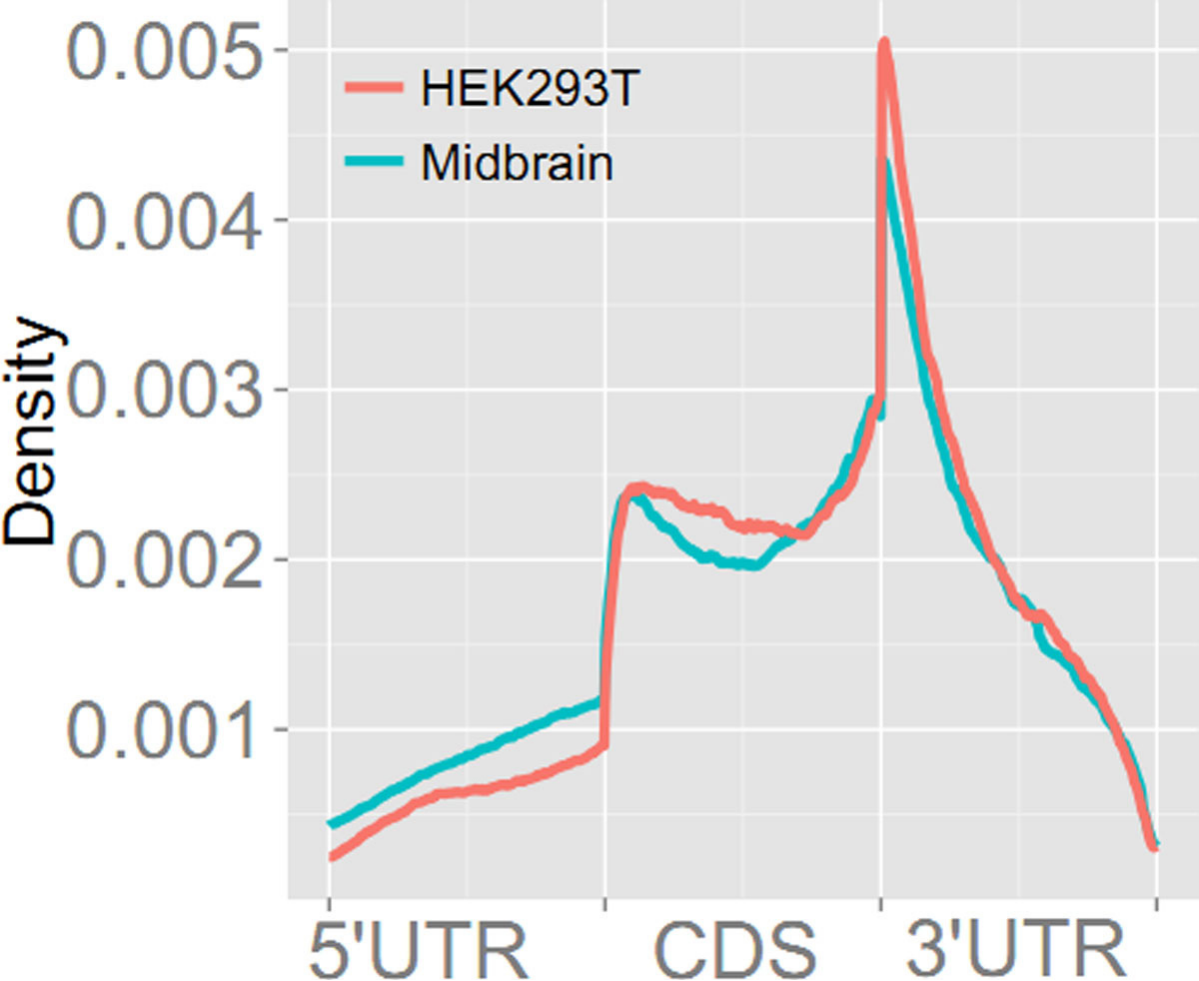
Distribution of m^6^A sites

**Figure 9:**
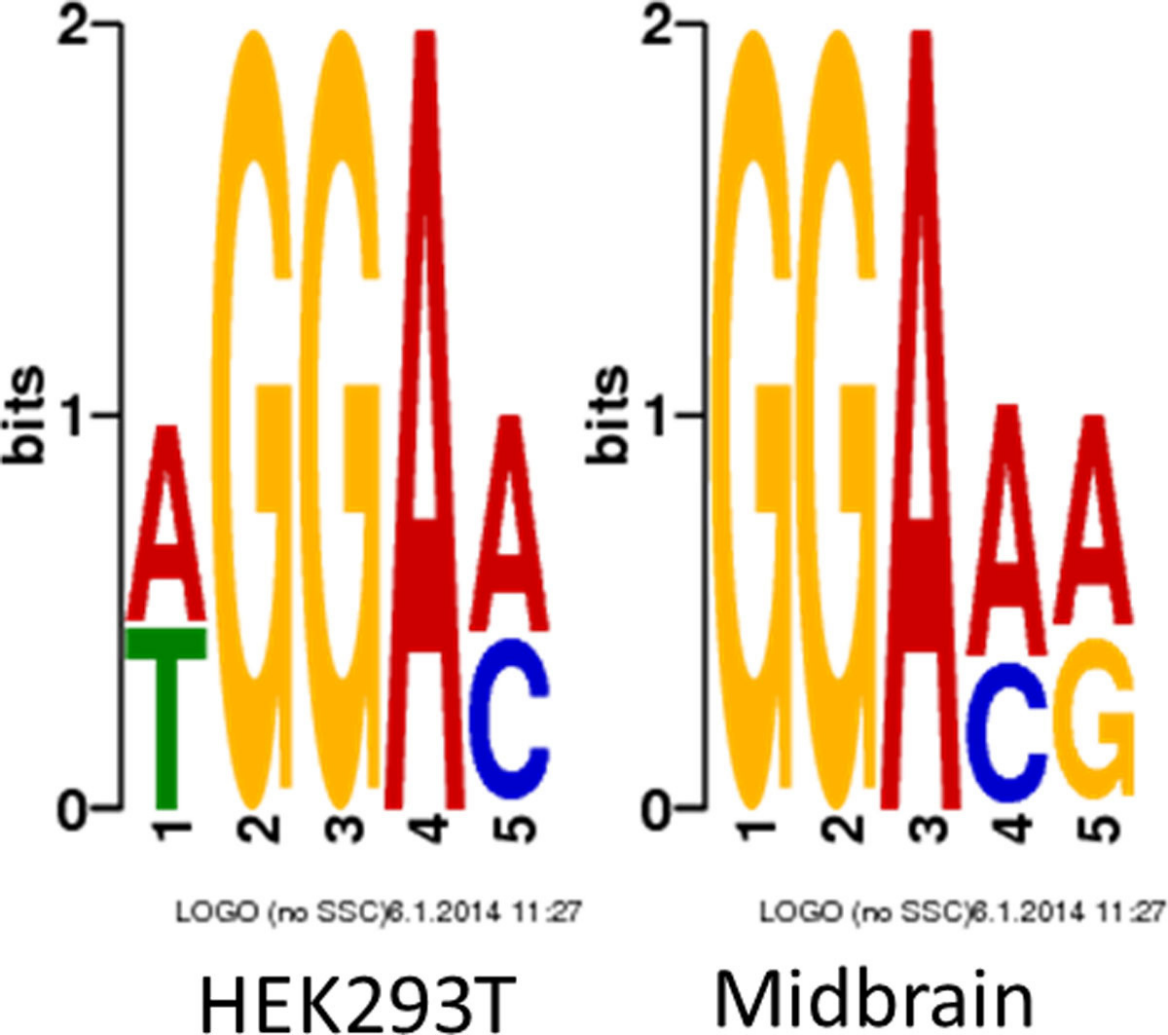
Motifs detected by DREME in human and mouse cells

On average, HEPeak predicted 2.27 and 2.22 sites per gene in human and mouse, respectively. Next, we examined the pattern of m^6^A sites by mapping all the peaks to the transcriptome and tallying the distribution of m^6^A sites in genes. For mRNA residing peaks, about 45% of the peaks located in the 3'UTRs, about 35% in the CDS, and only less than 20% from the 5'UTR (Figure 7). As shown in Figure 8, m^6^A methylation sites were significantly enriched near the stop codon and overly present in the 3'UTR for both human and mouse, indicating that m^6^A may be involved in transcriptional regulation, consistent with the reported results in previous studies [1,2]. To gain additional insights into prediction, DREME [26] was performed on the called peak sequences to predict the motif of the m^6^A methylation site. As shown in Figure 9, the most enriched motifs for the HEK293T cells and mouse midbrain cells are GGACH [10,11], which were identified bound by methytransferase METTL3 and METTL14 [27].

### HEPeak revealed distribution of m^6^A in lncRNA

**Figure 10:**
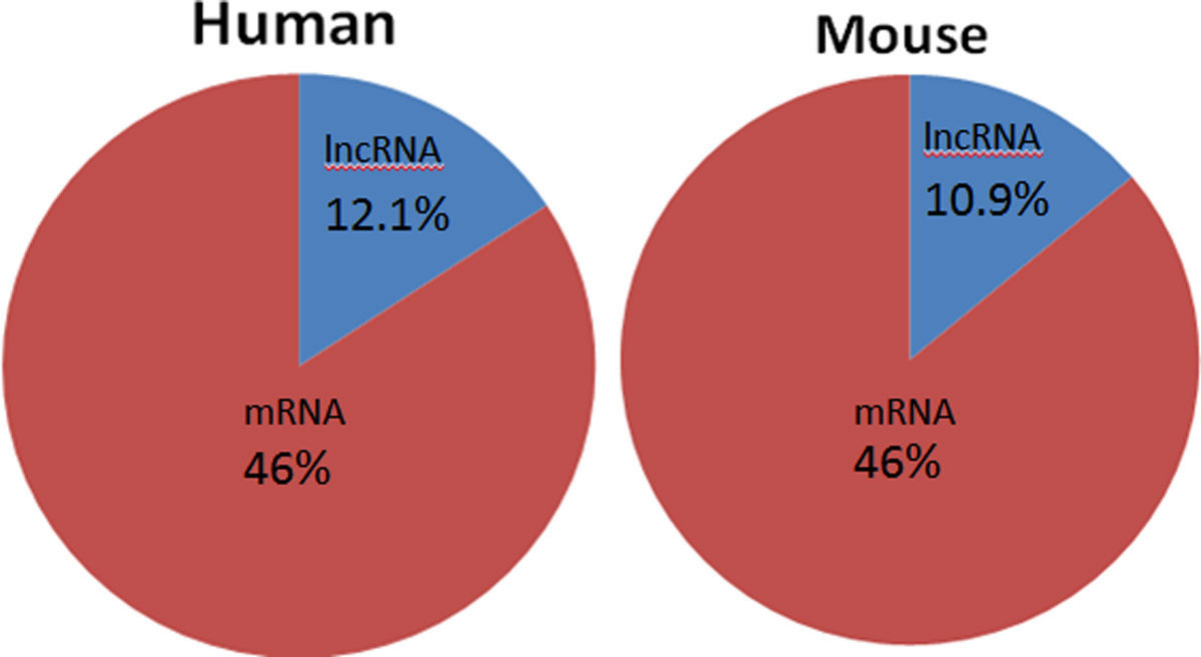
**Proportions of m^6^A occurrence in mRNAs and lncRNAs**.

**Figure 11:**
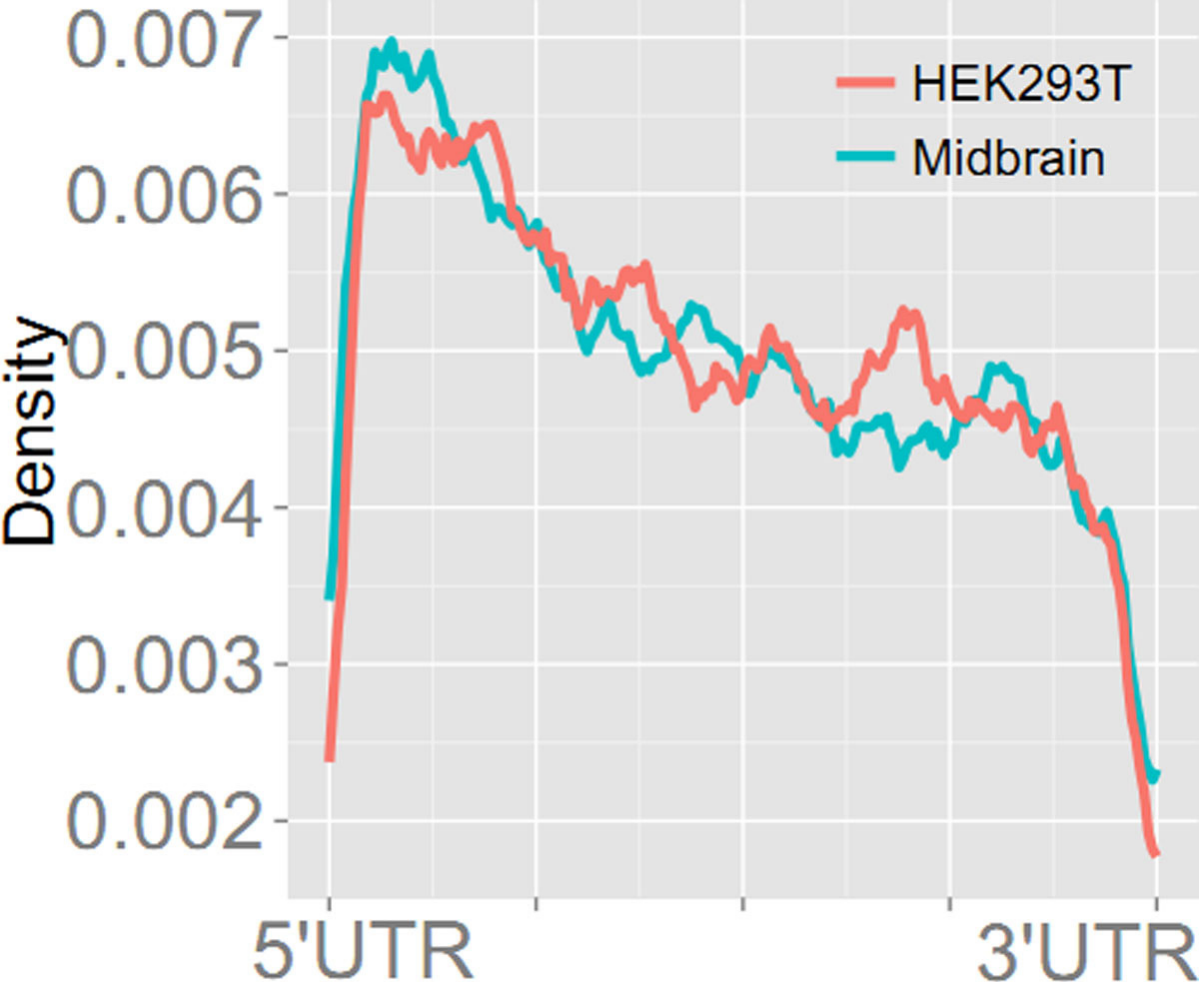
**Distribution of m^6^A in lncRNAs and mRNAs**.

We next examined the m^6^A sites predicted by HEPeak in long non-coding RNAs (lncRNAs), i.e., non-coding RNAs of more than 300 bp in length. m^6^A sites were found in lncRNAs in [28,29]. In human HEK293T cells, about 1847 peaks were predicted in lncRNAs, which accounted for 12.1% of the total predicted peaks (Figure 10). Similarly, in mouse midbrain cells, 2759 peaks (10.9% of the total peaks) were detected in lncRNAs. We then examined the distribution of the peaks in lncRNA in human HEK293T cells and found it is significantly different from that in mRNAs (Figure 11). Instead of being enriched near the stop codon in mRNAs, m^6^A sites in lncRNAs favour 5'UTR over 3'UTR. A similar pattern was also observed for mouse midbrain cells. These findings imply that the regulatory functions in mRNAs may be different from those in lncRNAs

## Conclusion

In this paper, a novel HMM-based peak calling algorithm, HEPeak, was developed for peak calling for MeRIP-seq data. By introducing the exome-based annotation, HEPeak circumvents the ambiguity related to isoforms. In order to characterize correlation between continuous bins in an m^6^A peak region, HEPeak utilized HMM to model the dependency. Additionally, IP reads and control reads are modelled in one mathematical model to avoid separate HMM peak-calling procedures in IP and control as in RIPSeeker [24]. Compared with ExomePeak, which treated each bin independently, HEPeak was shown to achieve higher detection specificity and sensitivity in the simulated data. When applying HEPeak to the collection of two published MeRIP-seq data from human and mouse, the results revealed that m^6^A methylation extensively existed in genes. HEPeak showed higher sensitivity than ExomePeak and predicted more novel m^6^A sites. Particularly, almost all the peaks detected by ExomePeak can be found by HEPeak. Moreover, with respect to the peak regions, m^6^A sites called by HEPeak were biologically more meaningful than ExomePeak, by connecting separate m^6^A sites together, of which gaps were not tested significantly enriched by ExomePeak due to the limitation of the independence assumption.

Furthermore, in both human and mouse mRNAs, the distributions of m^6^A sites were similar, where more m^6^A sites were observed in the 3'UTR as supposed to CDS and 5'UTR, and the sites were significantly enriched near the stop codon as previously reported. These findings highly suggest that m^6^A may play a role in transcriptional regulation. In addition, we examined the sequence motif of the predicted m^6^A sites and found that both human and mouse shared the similar m^6^A motif -GGACH. This consistency suggests that m^6^A methylation uses the same mechanism in different cells and species. Moreover, m^6^A sites were also predicted in lncRNAs but bear a different distribution from that in mRNAs, implying that m^6^A may have different roles in regulating mRNAs and lncRNAs.

## Appendix

The derivation of the EM solution is detailed in the following. Based on the notations defined in the main text, the total likelihood in the *m_th _*step of HEPeak is expressed as follows

(17) $$\begin{array}{l} Q(\theta^{\left(m-1\right)},\theta )=\sum_{z}p(Z|X,\theta^{\left(m-1\right)}*\ln p(X,Z|\theta )\\ =\sum_{z}p(Z|X,\theta^{\left(m-1\right)}*[\sum_{K}z_{1,k}*\ln\pi_{k}+\sum_{n=2}^{N}\sum_{j=1}^{2}\sum_{k=1}^{2}z_{n-1,j}z_{n,k}*\ln A_{j,k}\\ +\sum_{n=1}^{N}\sum_{k=1}^{2}z_{n,k}*\ln p(x_{n}|z_{n,k})] \end{array}$$

As defined in (7-8),

(18) $$\begin{array}{c} \underset{z}{\sum }p\left(Z|X,\theta \right)*z_{n,k}=\gamma \left(z_{n,k}\right)=E\left(z_{n,k}\right)\\ \underset{z}{\sum }p\left(Z|X,\theta \right)*z_{n-1,j}z_{n,k}=\varepsilon \left(z_{n-1,j},z_{n,k}\right)=E\left(z_{n-1},z_{n,k}\right) \end{array}$$

Given *x_n _*follows a binomial distribution, then

(19) $$\begin{array}{c} p\left(x_{n}|z_{n,k};t_{n}\right)=\left(\begin{array}{c} t_{n}\\ x_{n} \end{array}\right)*{p_{k}}^{x_{n}}{\left(1-p_{k}\right)}^{t_{n}-x_{n}}\\ \Leftrightarrow \text{ln}p\left(x_{n}|z_{n,k};t_{n},p\right)=\text{ln}t_{n}!-\text{ln}x_{n}!-\text{ln}y_{n}!\\ +x_{n}*\text{ln}p_{k}+\left(t_{n}-x_{n}\right)*\text{ln}\left(1-p_{k}\right) \end{array}$$

Thus, *p_k _*can be computed through maximizing the likelihood function of the total probability, the same as setting the first derivative equal to zero,

(20) $$\frac{\partial Q}{\partial p_{k}}=0\Rightarrow p_{k}=\frac{\overset{N}{\underset{n=1}{\sum }}\gamma \left(z_{n,k}\right)*x_{n}}{\overset{N}{\underset{n=1}{\sum }}\gamma \left(z_{n,k}\right)*t_{n}}$$

In the same fashion, *π_k _and A_j,k _*can be computed,

(21) $$\frac{\partial Q}{\partial \pi_{k}}=0\Rightarrow \pi_{k}=\frac{\gamma \left(z_{11}\right)}{\overset{2}{\underset{j=1}{\sum }}\gamma \left(z_{1j}\right)}$$

(22) $$\frac{\partial Q}{\partial A_{j,k}}=0\Rightarrow A_{j,k}=\frac{\overset{N}{\underset{n=2}{\sum }}\varepsilon \left(z_{n-1,j},z_{n,k}\right)}{\overset{2}{\underset{l=1}{\sum }}\overset{N}{\underset{n=2}{\sum }}\varepsilon \left(z_{n-1,j},z_{n,l}\right)}$$

## List of abbreviations used

HMM, Hidden-Markov Model; FDR, False discovery rate; HEPeak, HMM-based exome peak calling method; ExomePeak, Exome-based peak calling method; MeRIP-seq, Methylated RNA Immunoprecipatation combined with RNA sequencing; EM, Expectation of maximum likelihood method; CDS, Coding DNA sequence; UTR, Untranslated region.

## Competing interests

The authors declare that they have no competing interests.

## Authors' contributions

XC and YH designed the method and drafted the manuscript. JM helped with preprocessing the data and analyzed the peak distribution. MKR and CY provided biological interpretation of results on real data. YH supervised the work, made critical revisions of the paper, and approved the submission of the manuscript.

## Declarations section

Publication of this article was supported by National Science Foundation (CCF-0546345) to YH.

This article has been published as part of *BMC Genomics *Volume 16 Supplement 4, 2015: Selected articles from the IEEE International Workshop on Genomic Signal Processing and Statistics (GENSIPS) 2013. The full contents of the supplement are available online at http://www.biomedcentral.com/bmcgenomics/supplements/16/S4.
